# Renoprotective effects of *Andrographis paniculata* (Burm. f.) Nees in rats

**DOI:** 10.1080/03009730903174321

**Published:** 2009-09-07

**Authors:** Pratibha Singh, Man Mohan Srivastava, Lakhu Dev Khemani

**Affiliations:** ^1^Department of Chemistry, Dayalbagh Educational Institute (Deemed University)AgraIndia

**Keywords:** Albino rats, andrographis, aqueous extract, ayurveda, blood urea nitrogen, nephrotoxic, renal failure, serum creatinine, serum urea, wistar rats

## Abstract

**Background:**

Renal failure is an increasingly common condition with limited treatment options that is causing a major financial and emotional burden on the community. *Andrographis paniculata* is the plant used in Ayurveda for several remedies. Scientific evidence suggests its versatile biological functions that support its traditional use in the Orient. The plant is claimed to possess immunological, antibacterial, anti-inflammatory, antithrombotic, and hepatoprotective properties. But, to date, there is no study demonstrating the protective effect of *A. paniculata* on gentamicin-induced renal failure. The present study aims to highlight the first ever reported, antirenal failure activity of *A. paniculata*.

**Methods:**

Male Wistar albino rats were divided into three groups: normal control, gentamicin control, and aqueous extract of *A. paniculata* (200 mg/kg, per oral (p.o.))-treated. The nephrotoxic model was induced by gentamicin (80 mg/kg, intraperitoeal (i.p.)). Blood samples were examined for serum creatinine, serum urea, and blood urea nitrogen after the 10 days of treatment.

**Results:**

A gentamicin-induced nephrotoxic animal model was successfully prepared. Aqueous extract of *A. paniculata* attenuated the gentamicin-induced increase in serum creatinine, serum urea, and blood urea nitrogen levels by 176.92%, 106.27%, and 202.90%, respectively.

**Conclusion:**

The present study reports that the aqueous extract (whole plant) of *A. paniculata* (Burm. f.) Nees exhibits a significant renoprotective effect in gentamicin-induced nephrotoxicity in male Wistar albino rats.

## Introduction

Kidney disease is the ninth leading cause of death in the United States (1). The most recent data suggest that 27 million individuals have chronic kidney disease, representing nearly one in every seven adults and a 30% increase over the past decade (2). More than 200,000 people in the US suffer from kidney failure (3). The prevalence of non-terminal renal failure in Swedish children 1 to 15 years of age was registered as 4.50 per million total population (4). This increase is not unique to Sweden; many Asian countries have also seen a similar increase in the incidence of end-stage renal failure (ESRF) due to an increasing incidence of the risk factors for renal disease (5). Population-sampling studies from around the globe indicate similar prevalence rates, usually ranging between 10% and 13% (6. Two community-based studies, although methodologically different, have shown a prevalence of chronic renal failure of 0.16% and 0.79% in India (7). To date, renal replacement is the only therapy for ESRF. In case of non-availability of a kidney, dialysis is the only alternative, which unfortunately is severely limited by several constraints including a good amount of expenditure. No exclusive drug has been reported so far in any category of medical treatment. The popularity of complementary medicine has increased worldwide. Herbal remedies have been developed through traditional knowledge of herbs, which is a ray of hope for kidney failure patients. A number of herbs, traditionally used, are *Phoenix dactylifera* (8), *Kalanchoe pinnata* (9), *Ficus exasperata* (10), and *Curcumin* (11). In continuation of our work exploring herbal potential for diabetes (12,13) and antioxidants 14, the present paper explores the potential of aqueous extract of *Andrographis paniculata* for significant amelioration of renal failure in gentamicin-induced nephrotoxic male Wistar albino rats.

*A. paniculata* (Burm. f.) Nees (Acanthaceae), commonly known as ‘kalmegh’ in India, is used as a bitter ingredient in 26 Ayurvedic formulations as immunomodulatory (15), antiangiogenic (16), anticancer (17), and in treatment of various liver disorders (18). However, no attention has been paid so far to exploring its renoprotective activity in animals and human beings.

## Materials and methods

### Plant material

The fresh whole plants of *A. paniculata* properly identified and authenticated were obtained from the National Botanical Research Institute, Lucknow, India. These were washed, dried, powdered, and finally stored in air-tight glass jars.

### Preparation of extract

The whole plant material (5.0 g) was extracted with distilled water (100 mL) twice, overnight at 28±2°C with continuous stirring. The extract was evaporated under reduced pressure to a known volume (100 mL) and was evaporated to dryness at 40°C. The strength of the aqueous root extract was calculated to be 50 mg/mL.

### Animal model

The study was performed on male Wistar albino rats of approximately the same age-group and body-weight (2–3 weeks; 130±10 g), housed in ventilated animal rooms at a temperature of 24±2°C with a 12 h light/dark cycle and 60%±5% humidity. They were fed with Amrut Laboratory Animal Feed, manufactured by Nav Maharashtra Chakan Oil Mills Ltd, Pune, India. Water was provided *ad libitum*. All experiments were performed according to the norms of the local ethical committee.

### Experimental protocol

Animals were randomly divided into three groups of five animals each. Group I was kept as normal control receiving isotonic saline (0.5 mL, i.p.) for 8 consecutive days, and animals of groups II and III were administered gentamicin, manufactured by Wockhardt Ltd, India (80 mg/kg/day, i.p.) for 8 consecutive days, which is well known to produce significant nephrotoxicity in rats (19). Injections of gentamicin were made daily at 08:00 hours to minimize the circadian variation in nephrotoxicity (20).

Groups I and II received vehicle (0.5 mL gum acacia, p.o.) for 10 days and group III was orally administered aqueous extract of *A. paniculata* suspended in 1% w/v gum acacia at a dose of 200 mg/kg for 10 days. After the last application, individual rats were placed in metabolic cages for 24 hours of urine collection to determine the urine output. At the end of 24 hours, the rats were anaesthetized with a combination of ketamine (60 mg/kg) and xylazine (5 mg/kg) given intraperitoneally. Blood samples were collected via retro-orbital puncture in plain plastic tubes, left to stand at 4°C for 1 hour, and centrifuged (900 *g* for 15 min at 5°C) to separate serum. The serum obtained was stored at −5°C until analysis.

### Biochemical analysis

The concentration of creatinine in serum was measured by Jaffe reaction using a commercial kit (Human, Germany). The urea level was measured in serum by using a commercial kit (Beacon Diagnostics, India), and blood urea nitrogen concentration was measured by using a commercial kit (ERBA Diagnostics, Germany).

### Statistical analysis

All results are expressed as mean±standard error (SE). Statistical differences between correlated samples were evaluated using Student's *t* test and noted to be significantly different where *P*<0.05. Student *t* test calculations were assessed on the online scientific calculators (21). Composite treatments were compared using one-way analysis of variances (ANOVA) and considered significantly different where probability values were found to be equal to or less than 0.05. All ANOVA tests, as well as mean and standard error of mean calculations, were performed using GraphPad Prism (GraphPad Software, Inc., San Diego, USA).

## Results

Gentamicin (80 mg/kg) when injected for 8 consecutive days caused marked nephrotoxicity as is evident from Table I, showing significant (*P* < 0.001) increase in serum creatinine (196.92%), serum urea (121.96%), and blood urea nitrogen (241.65%) as compared to normal control animals. These results were supported by 24 h urine output of individual nephrotoxic rats of group II. The urine volume of nephrotoxic animals was found to be significantly (*P* < 0.001) increased (by 106.20%) when compared to normal control. The aqueous extract of *A. paniculata*-treated rats differed from normal control rats by an elevated concentration of serum creatinine (20.00%, *P* > 0.05), serum urea (15.69%, *P* < 0.01), blood urea nitrogen (38.75%, *P* < 0.0001), and urine volume (15.50%, *P* > 0.05). No statistical difference could be obtained in serum creatinine and urine volume, while the levels of blood urea nitrogen and serum urea were found to be statistically significantly different as compared to normal rats. Hence, we conclude that all the parameters in the extract-treated group were not identical but closer to the normal animals. The extent of protection offered by the test extract was found to be 176.92%, 106.27%, and 202.9% in terms of biochemical parameters, namely serum creatinine, serum urea, blood urea nitrogen, respectively, and the 90.70% reduction in urine volume voided also supports the amelioration of polyuric acute renal failure by the aqueous extract of *A. paniculata*.

**Table I.** Effect of aqueous extract of *Andrographis paniculata* (200 mg/kg) on serum creatinine, serum urea, blood urea nitrogen levels and urine volume in gentamicin-induced nephrotoxic rats.

Groups	Serum creatinine (µmol/L)	Serum urea (mmol/L)	Blood urea nitrogen (mmol/L)	Urine volume (L)
Normal	57.46±0.01	5.25±1.03	6.89±1.40	2.58×10^−3^±0.11
Gentamicin control (80 mg/kg, i.p.)	170.61±0.30^a^ (196.92)	11.64±0.43^b^ (121.96)	23.54±0.63^b^ (241.65)	5.32×10^−3^±0.20^b^ (106.20)
Extract-treated (200 mg/kg, p.o.)	68.95±0.10^c^ (20.00)	6.07±0.32^d^ (15.69)	9.56±0.16^d^ (38.75)	2.98×10^−3^±0.50^c^ (15.50)
Statistics (one-way ANOVA)	*F*=15.607 (*P*<0.001)	*F*=44.690 (*P*<0.001)	*F*=42.977 (*P*<0.001)	*F*=23.622 (*P*<0.001)

Values are mean±SE (*n*=5). Data for normal animals are considered as base-line data; there was no significant base-line difference between the groups. Percentage increase (in parentheses) is calculated with reference to normal control. ^a^*P*<0.01 versus control group. ^b^*P*<0.001 versus control group. ^c^*P*<0.01 versus gentamicin group. ^d^*P*<0.001 versus gentamicin group.

## Discussion

This study strongly suggests that aqueous extract of *A. paniculata* exhibits a protective action on gentamicin-induced acute renal failure. Gentamicin has a nephrotoxic potential as indicated by the impairment in the renal function, which was reflected in the significant increases in serum creatinine, serum urea, and blood urea nitrogen levels when compared with the normal controls (Table I). It has been documented that gentamicin-induced nephrotoxicity resulting in an increase in serum creatinine, accompanied by an increase in blood urea nitrogen levels and severe proximal renal tubular necrosis, leads to renal failure (22,23). The significant increases in urine output recorded in the present study also confirm the gentamicin-induced polyuric acute renal failure.

The effects induced by gentamicin were significantly reversed by the aqueous extract of *A. paniculata*, adding further evidence that this plant has the potential to be used to ameliorate gentamicin nephrotoxicity.

Gentamicin administration to rats appears to enhance the production of O_2_^–^ anions and unstable OH^•^ radicals. Andrographolide, one of the major chemical constituents of the plant *A.* *paniculata*, has been reported to possess antioxidant activity (24). It may be hypothesized that gentamicin treatment is likely to produce free radicals indicating oxidative damage at the cellular level of the renal cortex. Thus, the possible mechanism of renoprotection of *A.* *paniculata* may be attributed to its antioxidant and free radical-scavenging properties. The exact mechanism by which *A. paniculata* extract ameliorates gentamicin-induced nephrotoxicity remains to be elucidated.

In conclusion, the present study explores for the first time that the aqueous extract of *A. paniculata* was able to produce considerable alleviation from the nephrotoxic action of gentamicin in male albino rats and hence exhibits marked renoprotective activity and can be deemed to be a good bioagent for the treatment of acute renal injury induced by nephrotoxins. Chromatographic fractionation of different parts of the plant extracted in a series of organic solvents, followed by characterization of the bioactive principle(s) responsible for the observed significant renoprotective efficacy of the plant, is in progress.
